# Intramuscular Botulinum toxin A injections induce central changes to axon initial segments and cholinergic boutons on spinal motoneurones in rats

**DOI:** 10.1038/s41598-020-57699-z

**Published:** 2020-01-21

**Authors:** D. B. Jensen, S. Klingenberg, K. P. Dimintiyanova, J. Wienecke, C. F. Meehan

**Affiliations:** 1 Department of Neuroscience, University of Copenhagen, Panum Institute, Blegdamsvej 3, DK-2200 Copenhagen, Denmark; 2 Department of Nutrition, Exercise and Sports, University of Copenhagen, Nørre Allé 51, DK-2200 Copenhagen, Denmark

**Keywords:** Neuroscience, Neurology

## Abstract

Intramuscular injections of botulinum toxin block pre-synaptic cholinergic release at neuromuscular junctions producing a temporary paralysis of affected motor units. There is increasing evidence, however, that the effects are not restricted to the periphery and can alter the central excitability of the motoneurones at the spinal level. This includes increases in input resistance, decreases in rheobase currents for action potentials and prolongations of the post-spike after-hyperpolarization. The aim of our experiments was to investigate possible anatomical explanations for these changes. Unilateral injections of Botulinum toxin A mixed with a tracer were made into the gastrocnemius muscle of adult rats and contralateral tracer only injections provided controls. Immunohistochemistry for Ankyrin G and the vesicular acetylcholine transporter labelled axon initial segments and cholinergic C-boutons on traced motoneurones at 2 weeks post-injection. Soma size was not affected by the toxin; however, axon initial segments were 5.1% longer and 13.6% further from the soma which could explain reductions in rheobase. Finally, there was a reduction in surface area (18.6%) and volume (12.8%) but not frequency of C-boutons on treated motoneurones potentially explaining prolongations of the after-hyperpolarization. Botulinum Toxin A therefore affects central anatomical structures controlling or modulating motoneurone excitability explaining previously observed excitability changes.

## Introduction

Botulinum neurotoxin type-A (BoNT/A) is one of the most dangerous toxins known to man, yet it is being increasingly used, both cosmetically and clinically, to produce long lasting paralysis of specific muscles. After intramuscular injection, the toxin enters the synaptic vesicles of motor axon terminals by endocytosis. From here it silences synaptic transmission via specific proteolytic cleavage of SNAP-25 which prevents the release of acetylcholine from the motoneurone terminal resulting in flaccid paralysis^1^. There is increasing evidence, however, that the effects may not be restricted to the periphery. Studies show that BoNT/A, injected intramuscularly, is transported both anterogradely along sensory axons and retrogradely along motoneurone axons to the motoneurone soma in the spinal cord^2–5^. Furthermore, there is now also evidence that BoNT/A can spread between networks of cells^6^, although this claim has been questioned by other studies and would appear to be dose dependent^7,8^. This raises the question as to what central effects BoNT/A may have on the motoneurone itself or on central cholinergic synapses contacting the motoneurone.

Electrophysiological studies suggest changes in spinal motoneurone excitability after intramuscular BoNT/A injections of the gastrocnemius muscle. Increased stretch reflexes are seen at two weeks post-injection in rats^9^. This increased reflex was also seen when tested as simple monosynaptic responses to dorsal root stimulation suggesting the enhanced reflexes may be due to an increase in motoneurone excitability^10^. Consistent with this, intracellular recordings of gastrocnemius motoneurones show a reduction in rheobase currents for action potentials already by 5 days post-injection^11^ which is further reduced by 2–3 weeks^12^. There are a number of potential anatomical factors that could account for these changes. The first is a possible reduction in cell size. This is plausible given that increases in input resistance have been observed following BoNT/A treatment^12^. Plasticity at the site of action potential generation; the axon initial segment (AIS), is another possible explanation. The size and location of the AIS determines the excitability of a neurone and its response to inputs^13^. This structure is highly plastic, and can dynamically change its size and location to alter neuronal excitably in response to perturbations in inputs or activity levels^14–16^. Thus, AIS plasticity would also appear to be a likely candidate for reducing the rheobase after peripheral BoNT/A injection.

A prolongation of the post-spike after-hyperpolarisation (AHP) of gastrocnemius motoneurones has also been observed at 2 weeks post BoNT/A injection^12^. A certain type of cholinergic synapse on spinal motoneurones; the C-bouton, plays a crucial role in the modulation of the motoneurone AHP of lumbar spinal neurones via their interaction with muscarinic (m2) receptors and the small conductance calcium-activated potassium (SK) channels mediating the AHP^17,18^. A reduction in the number and size of C-boutons on spinal motoneurones has been shown on axotomised motoneurones^19–21^ which may explain the prolongation of the AHP observed after axotomy^22,23^. As BoNT/A injections induce similar (albeit less extreme) changes in the AHP^12^ this suggests that a loss of functional connectivity at the neuromuscular junction may be enough to drive such changes in C-boutons. Alternatively, if BoNT/A is transported retrogradely and transsynaptically this could also influence the C-boutons.

**Figure 1:**
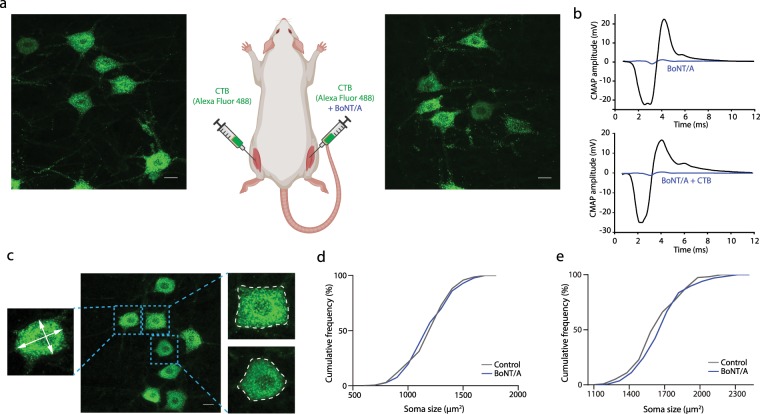
Intramuscular BoNT/A injection does not affect motoneurone soma size. (**a**) Maximum projections from 50 µm thick confocal stacks showing examples of traced motoneurones from rats injected with Cholera toxin subunit B (CTB) conjugated with Alexa Fluor 488 into gastrocnemius muscle on one side and CTB mixed with BoNT/A into the contralateral gastrocnemius (rat illustration created with BioRender.com). (**b**) Examples of maximal Compound Muscle Action Potentials (CMAPs) measured through the skin directly on top of the gastrocnemius muscle whilst stimulating the sciatic nerve at supramaximal stimulation. Recordings were performed bilaterally before (black traces) and 4 days after BoNT/A injections (blue traces). On one side BoNT/A only was injected (top panel), while the other side was injected with BoNT/A mixed with the CTB tracer (lower panel). As can be seen, a drastic reduction in CMAP amplitude occurred on both sides. (**c**) Confocal stack as described above showing the variability of soma shapes of traced neurones and the two methods used to estimate soma size. This was either by measuring the maximum and minimum diameter (left) and using these to calculate the area of an ellipse or by measuring the perimeter (right). The relative frequency distributions of the data for the measurements using the first and second methods are shown in figure (**d**,**e**) respectively. From these it can be seen that there were no significant differences in the soma size using the maximum and minimum diameter method (Control: n = 170, mean = 1224 µm^2^, SD = 195; BoNT/A: n = 186, mean = 1225 µm^2^, SD = 203.5; Unpaired T-test. P = 0.9908). Similarly, there was no significant differences when the perimeter measurement was used (Control: n = 118, mean = 1683.5 µm^2^, SD = 202.8; BoNT/A: n = 135, mean = 1685.7 µm^2^, SD = 204.1). Scale bars (**a**,**c**) 25 µm.

We therefore investigated possible intramuscular BoNT/A injection-induced anatomical changes in cell size, axon initial segment size/location and size/frequency of C-boutons on gastrocnemius motoneurones that could explain the altered excitability. BoNT/A was mixed with a tracer, Cholera toxin subunit B (CTB) before being injected unilaterally into the gastrocnemius muscle and CTB only injections on the contralateral side provided an internal control (Fig. 1a).

## Results

To determine that mixing the BoNT/A with tracer did not impair its effects, in 3 additional rats we recorded the maximal Compound Muscle Action Potential (CMAP) from the gastrocnemius muscles before and after bilateral injections of BoNT/A with CTB mixed with the BoNT/A on one side only. In all rats, by 4 days post-injection a drastic reduction in CMAP amplitude was observed on both sides (>75%) regardless of whether the BoNT/A had been mixed with tracer or not (Fig. 1b) confirming that CTB did not impair the ability of the BoNT/A to block neuromuscular transmission. The effects of BoNT/A on soma size, AIS parameters and C-boutons were then investigated using bilateral injections of CTB which on one side only was mixed with BoNT/A in 7 other rats.

### BoNT/A does not affect soma size

Examples of traced gastrocnemius motoneurones for both the BoNT/A injected and contralateral control side can be seen in Fig. 1a. The mean 2-D area was calculated using the maximum and minimum diameter of an ellipse fitted inside the soma (Fig. 1c,d). As there exists considerable variability in soma size of spinal motoneurones within animals (due to the different functional classes of motoneurones) the overall mean and distribution of soma size across all rats was compared which was not significantly different between the BoNT/A treated side and the control side (Unpaired t-test, t = 0.01149, df = 354, P = 0.9908, F test to compare variance, F = 1.090, P = 0.5702, Fig. 1d). This was then further confirmed by calculating the mean soma size for individual rats for the treated and untreated sides which were also not significantly different (Wilcoxon signed ranks test: sum of signed ranks = −8, n = 7 rats, P = 0.5781).

As motoneurone soma shapes varied considerably (Fig. 1c) we confirmed these findings by an additional analysis. Here, the soma size in the 2D plane was calculated by delineating the perimeter for each motoneurone (as shown in Fig. 1c). The soma size measured with this method was still not significantly different between the BoNT/A treated side and the control side when analysed by cell (Mann Whitney: U = 0.9993, P = 0.0999, Fig 1e) or using the mean per animal (Wilcoxon signed ranks test, n = 7 rats, P > 0.999). We can conclude therefore that at 2 weeks post-injection BoNT/A has no effect on the soma size of the affected motoneurones.

### BoNT/A results in an increase in length and distal relocation of motoneurone AISs

**Figure 2:**
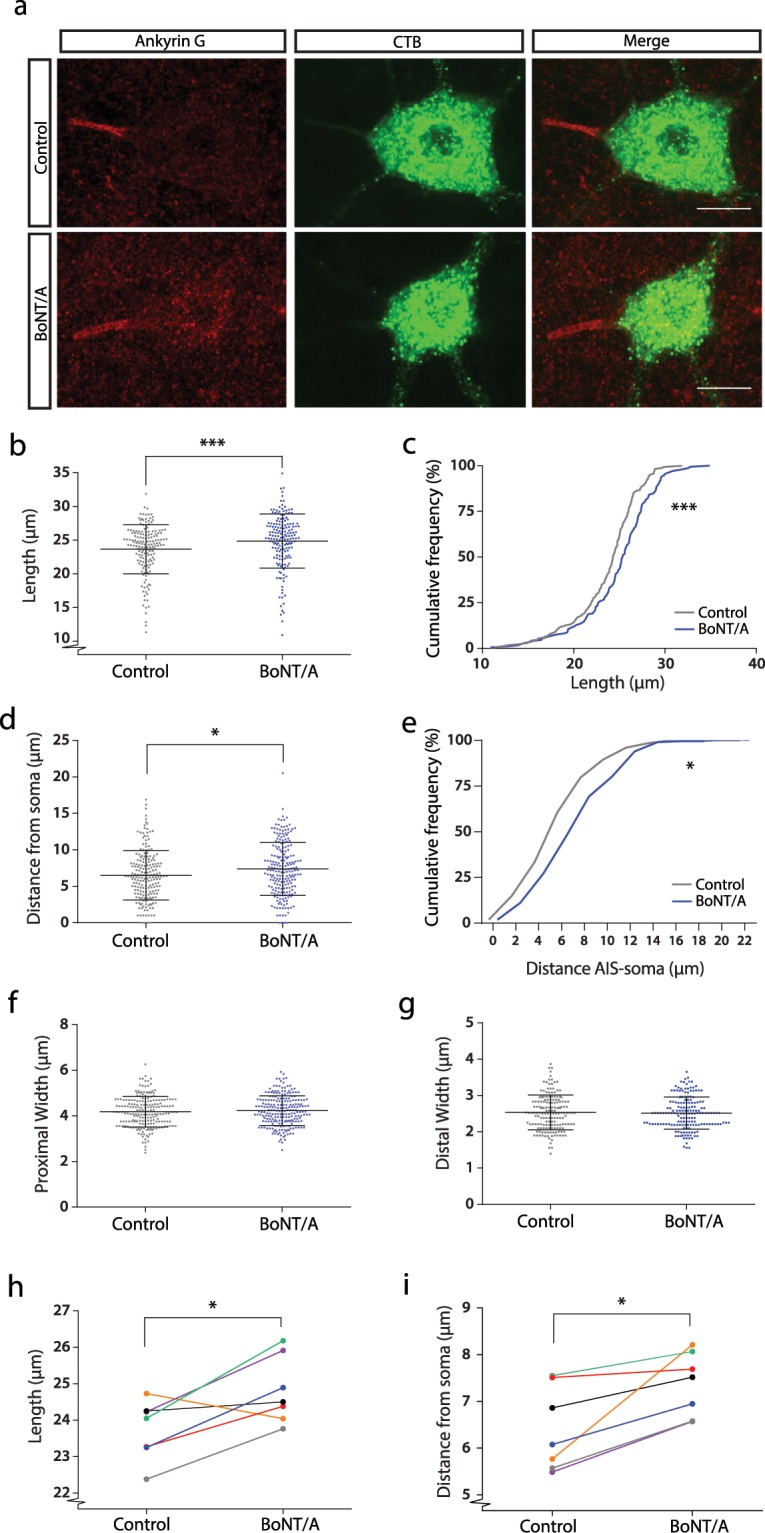
BoNT/A injection results in longer AIS’s that are further away from the soma. (**a**) Maximum projections from 50 µm thick confocal stacks showing examples of traced motoneurones (green) from both the control and BoNT/A injected side with axon initial segments labelled with antibodies against Ankyrin G (red). A scatter dot plot showing the individual data points, mean and standard deviations for length measurements of the AIS’s is shown in (**b**) and the cumulative frequency plot for this data is shown in (**c**) showing that AISs were significantly longer on the BoNT/A injected side (Control: n = 167, mean = 23.64 µm, SD = 3.63; BoNT/A: n = 172, mean = 24.85, SD = 4.01; Mann Whitney test. P = 0.0008). Similar graphs for measurements of the distance of the AIS from the soma are shown in (**d**,**e**) from which it can be seen that AISs are significantly further from the soma on the BoNT/A treated side (Control: n = 201, mean = 6.51 µm, SD = 3.37; BoNT/A: n = 215, mean = 7.40 µm, SD = 3.63; Mann Whitney test. P = 0.0106). Scatter dot plots showing the individual data points, mean and standard deviations for AIS proximal and distal width are shown in figure (**f**,**g**) where no significant differences can be seen for either proximal width (Control: n = 201, mean = 4.18 µm, SD = 0.67; BoNT/A: n = 222, mean = 4.23 µm, SD = 0.65; Unpaired t test. P = 0.4954) or distal width (Control: n = 167, mean = 2.53 µm, SD = 0.48; BoNT/A: n = 172, mean = 2.52 µm, SD = 0.44; Unpaired t test. P = 0.7264). The means for individual rats for AIS length can been seen in the graph in (**h**), showing that the result was mostly consistent across animals (n = 7 rats. Control: mean = 23.75, SD = 0.92; BoNT/A: mean = 24.82, SD = 0.80; Wilcoxon test. P = 0.0469). A similar graph showing the individual rat means for distance of the AIS from the soma shows a consistent effect across all rats (n = 7 rats. Control: mean = 6.40 μm, SD = 0.89; Botox: mean = 7.37, SD = 0.68; Wilcoxon test. P = 0.0156). Scale bar (a) 25 µm.

Immunohistochemistry for Ankyrin G was used to label AISs on traced gastrocnemius motoneurones as this scaffolding protein spans the entire length of the AIS^24^. Examples of AISs from both BoNT/A treated and control gastrocnemius motoneurones can be seen in Fig. 2a. No obvious abnormalities in gross morphology of the AIS were observed following BoNT/A injections, however, subtle changes in the length and location of AISs of gastrocnemius motoneurones were observed. As can be seen from the scatter dot plots in Fig. 2 there exists a large range with respect to AIS parameters that exists even within animals. Due to this, we first analysed the data by cell rather than by animal. AISs were significantly longer on treated neurones than on controls by 5.1% (Mann Whitney, P = 0.0008, Fig. 2b). This was due to a general shift in the BoNT/A treated population towards longer AIS (Fig. 2c). AISs were also further away from the soma (by 13.6%) on the BoNT/A side (P = 0.0106, Fig. 2d,e) but no changes were observed with respect to the proximal or distal width of the AIS (Fig. 2f,g).

To determine whether the observed differences were consistent across rats, means were calculated per animal and these compared using repeated-measures tests. This showed that the increase in length was consistent in 6 out of 7 rats (Wilcoxon signed ranks test: n = 7 rats, sum of signed ranks = −24, P = 0.0469, Fig. 2h), although in 1 rat this was a relatively minor change. The increase in the distance from the soma was observed in all 7 rats (Wilcoxon signed ranks test: n = 7 rats, sum of signed ranks = −28, P = 0.0156, Fig. 2i). Of interest, when the data is presented by rat, there is one outlier which showed a decrease in AIS length (orange data point, Fig. 2h). AISs from this particular animal also showed a much greater distal relocation than the other rats (Fig. 2i). This one rat therefore showed changes more consistent with axotomy (unpublished data). One explanation could therefore be possible axon damage during the surgery in this rat.

Although the use of the contralateral (non-BoNT/A treated side) has the advantage of being an internal control, eliminating the effects of inter-rat variation, it is possible that the BoNT/A could have contralateral effects (see discussion). To test this, we compared the mean AIS length on the CTB only side with the mean AIS length measured in an additional 5 control rats in which only CTB was injected. The mean AIS length in these extra control rats was 23.5 µm which was remarkably close to the mean of 23.6 µm measured in the control side of the BoNT/A treated rats and was not significantly different (P > 0.9999, Kruskal-Wallis, Post-hoc: Dunn’s multiple comparisons, n = 167 cells, contralateral control, n = 153 cells, naïve control). In fact, no significant differences were observed between the two different control groups with respect to any of the AIS parameters. A significant increase in AIS distance was still observed on the BoNT/A treated side when compared to these additional controls (P < 0.0001, Kruskal-Wallis, Post-hoc: Dunn’s multiple comparison, n = 215 cells, BoNT/A, n = 153 cells, naïve control).

### BoNT/A affects the size but not frequency of C-boutons on gastrocnemius motoneurones

**Figure 3:**
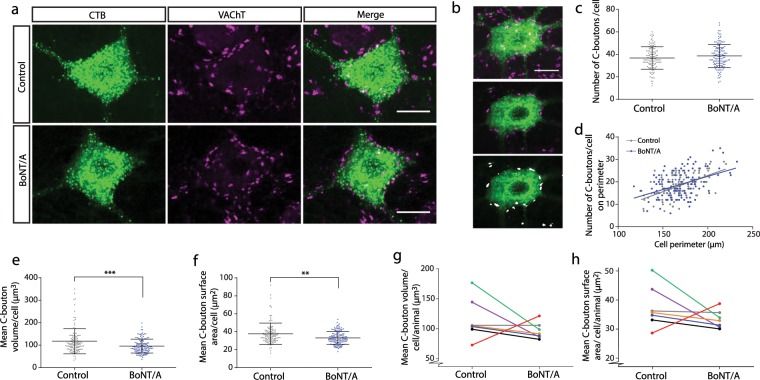
C-boutons are becoming smaller after BoNT/A injection. Maximum projections from 50 µm thick confocal stacks showing examples of traced motoneurones (green) with cholinergic boutons immunolabelled with VAChT (magenta) are shown in (**a**). In (**b**, top panel) the maximum projection from a 50 µm z-stack showing a traced motoneurone with C-boutons. In the middle panel the same cell is shown but, in this case, as a single confocal slice through the centre of the cell. In the lower image in the panel automated scripts in ImageJ were used to identify the C-boutons (labelled white). (**c**) shows a scatter dot plot showing the total number of C-boutons per cell along with means (and standard deviations) which was not different between the two groups (Control: n = 118, mean = 36.78, SD = 10.00; BoNT/A: n = 135, mean = 38.55, SD = 10.37). The number of C-boutons on the perimeter of the middle of the motoneurone (identified as shown in (b, lower panel)) can be seen plotted against the total perimeter of the cell in (**d**). There was no significant difference between the regression lines for the two sides (Control: n = 118; BoNT/A: n = 135; P = 0.4444). In (**e**) a scatter dot plot shows the individual cell values and the mean (and SD) for C-bouton volume per cell which was significantly decreased on the BoNT/A injected side (Control: n = 118, mean =  11.74 µm^3^, SD = 5.63; BoNT/A: n = 135, mean = 9.56 µm^3^, SD = 3.09; Mann Whitney test. P = 0.0008). In (**f**) a scatter dot plot shows values for individual cells along with means (and SD) for the C-bouton surface area which was also significantly decreased on the BoNT/A injected side (Control: n = 118, mean =  37.80 µm^2^, SD = 11.81; BoNT/A: n = 135, mean = 33.19, SD = 7.08; Mann Whitney test. P = 0.0011). Graphs (**g**,**h**) show the mean values for individual rats for the same data showing that the results are roughly consistent across rats but do not reach statistical significance for both C-bouton volume (n = 7 rats. Control: mean = 37.49 µm^2^, SD = 7.19; BoNT/A: mean = 33.32 µm^2^, SD = 3.09. Wilcoxon test. P = 0.1563) and surface area (n =  7 rats. Control: mean = 11.54 µm^3^, SD =  3.43; BoNT/A: mean = 9.64 µm^3^, SD = 1.36; Wilcoxon test. P = 0.1563). Scale bars (**a**,**b**) 25 µm.

C-boutons on the soma and proximal dendrites of spinal motoneurones were labelled using antibodies against vesicular acetylcholine transporter (VAChT). Examples of C-boutons labelled on BoNT/A treated and control motoneurones are shown in Fig. 3a. Automated scripts in Image J were used to identify, quantify and measure C-boutons on traced motoneurones (Fig. 3b). C-boutons are mostly restricted to the soma and proximal dendrites of motoneurones where they constitute the only cholinergic synapses^25,26^. Given that the number of proximal dendrites visible in a single 50 µm thick tissue section differed between cells we confined our analysis of C-boutons to the soma.

The mean number of C-boutons per cells was not significantly different between the BoNT/A treated and control sides (Unpaired t-test: t = 1.376, df = 251, P = 0.17, F test to compare variances: F = 1.074, P = 0.6923, Fig. 3c). As motoneurones are large cells it is possible that some of the boutons on the sides of the neurone facing the cut surface of the tissue may have been removed by the cutting process. To control for this, we also calculated the number of C-boutons around the perimeter of the cell in the centre of the soma (using the centre of the nucleus as a guide, Fig. 3b). Using the central perimeter measurement also allowed us to control for cell size as linear regression showed that C-bouton number increases with cell perimeter (p < 0.0001, Fig. 3d). The regression lines for C-bouton number by cell perimeter were not significantly different from each other (P = 0.37) confirming that BoNT/A does not cause a stripping of C-boutons on spinal motoneurones.

BoNT/A injection did, however, result in an 18.6% decrease of C-bouton volume (Mann Whitney test: U = 6024, P = 0.0008, Fig. 3e) and a 12.8% reduction of C-bouton surface area (Mann Whitney: U = 6061 test P = 0.001, Fig. 3f). To determine if this was consistent across rats the mean volume and surface area of C-boutons per cell was calculated for each animal. From this it could be seen that 5/7 rats show a reduction in C-bouton volume (Fig. 3g) and 6/7 show a reduction in C-bouton surface area, although it was not always a large difference and was not statistically significant (although we must express caution with this statistical comparison due to a low statistical power when analysing by rat).

## Discussion

The aim of these experiments was to investigate structural plasticity that could underlie the excitability changes occurring at 2 weeks following BoNT/A injections. Our most prominent observation was an increase in AIS length together with a distal relocation of the AIS. This distal relocation was not due to the increase in total length as the distance was measured from the soma to the most proximal border of the AIS. No differences in cell size were observed suggesting that these changes were not simply to compensate for any differences in electrical load due to soma size (although it must be noted that detailed measurements of dendritic trees were not possible). For cells with very large dendritic trees (such as spinal motoneurones) modelling suggests that excitability is increased by lengthening the AIS and moving it further away from the soma^27^. It could therefore be hypothesized that the distal relocation, together with the increase in length observed following BoNT/A would increase the excitability of the motoneurones. This would certainly be consistent with the decrease in rheobase^12^ and increased reflexes that have been shown following BoNT/A^9,10^.

An important question remains as to whether the magnitude of the AIS changes would be sufficient to have a functional consequence. Here modelling studies, based on AIS plasticity following blast induced traumatic brain injury have shown that reductions in AIS length of just 4.5% result in small increases in action potential threshold as well as decreased amplitude and rates of rise of action potentials. Importantly, it also reduced the firing frequency in response to prolonged depolarizing currents^28^. In the context of these results, we can speculate that both of our observed changes of a 5.1% increase in length and 13.6% distal relocation following BoNT/A are likely to have a functional consequence.

Changes in the size but not frequency of C-boutons were also observed on spinal motoneurones. Bouton size has been shown to systematically correlate with both total and individual active zone area, the number of active zones and the number of synaptic vesicles^29^. The reduction in size we observed would consequently reduce the effectiveness of the C-boutons. Given the role that C-boutons play in the reduction of the AHP via their modulation of SK channels^17,18^, this could explain the prolongation of the AHP observed in gastrocnemius motoneurones. One caveat here is that the role of C-boutons is hypothesised to be task dependent modulation, having the most effect under states of moderate to strong physiological drive^25,30^ and all recordings of AHP following BoNT/A treatment, however, have been performed in immobile anaesthetised animals.

It could be hypothesised that the AIS changes may result in a decrease in rheobase making the motoneurones easier to recruit and then once recruited the motoneurones would show increased firing rates. A prolongation of the AHP would therefore compensate to decrease firing rates, normalising the input-output function. From this perspective, the changes in C-boutons may potentially represent a homeostatic response to the AIS plasticity which may be particularly important under periods of intense drive to the motoneurones.

### Potential driving factors for AIS plasticity following BoNT/A injections

The increases in AIS length following BoNT/A treatment may itself represent a homeostatic response either to the loss of functional connectivity at the neuromuscular synapse or changes in inputs to the motoneurones, rather than a direct toxic effect from the BoNT/A itself as injury to motoneurones tends to result in AIS shortening (unpublished data). Reductions in excitatory sensory input to neurones have been shown to induce homeostatic increases in AIS length in auditory neurones^14^. One of the largest sources of excitatory sensory input to motoneurones are the Ia afferent synapses which mediate the stretch reflex. BoNT/A would alter this sensory input as the gamma motoneurone-intrafusal fiber connection also requires acetylcholine and is also blocked by intramuscular BoNT/A injections^31–34^. Such a block would result in a decreased sensitivity of the muscle spindles and consequently a reduced excitatory Ia input to the motoneurone which may trigger a homeostatic increase in AIS length in order to reduce rheobase to facilitate the afferent input reaching threshold. Ia afferent input may not be the only synaptic inputs to motoneurones that are affected. There are also experiments suggesting that after intramuscular injection, BoNT/A is not only retrogradely transported to the spinal cord^35^, but also travels transsynaptically and consequently may potentially be blocking other central synapses on the motoneurones^36^. Recent work extended on these findings showing that cleaved SNAP-25 can be visualised preferentially at cholinergic terminals on the soma of facial motoneurones following intramuscular BoNT/A injections^37^ suggesting that it is specifically blocking excitatory rather than inhibitory synaptic inputs to motoneurones. However, the question of whether, at clinically relevant doses, BoNT/A is actually transcytosed and the effects of this is a contentious issue. A recent study showed only sparse labelling of cleaved SNAP-25 in the spinal cord of rats following doses of BoNT/A at dosages close to those used in our experiments and suggested that the labelling seen in neighbouring neurones at higher dosages may be due to systemic spread^7^. In addition an *in vitro* study, using rat sympathetic neurones, suggested that while BoNT/A does migrate to the soma, no significant decrease in the amplitude of EPSPs recorded from the soma was seen at lower doses (but with significant decreases observed at much higher doses^8^). Further evidence to suggest that the reduction in C-bouton size observed in our experiments represents a homeostatic change rather than a toxic effect of the BoNT/A at the central cholinergic synapses are observations that the few central cholinergic synapses that had been shown previously to express cleaved SNAP-25 tend to increase in size due to vacuolisation in contrast to our observed general decrease in size^37^.

There is also limited (but again questioned^7^) evidence that the BoNT/A once transported to the spinal cord, can spread to the contralateral spinal cord^36^, or can enter the blood system and go systemic introducing a possible caveat to our work in using the contralateral motoneurones as internal controls. Our measurements of C-bouton size for another study exploring the effects of axotomy^38^, performed at the same time using all the same reagents and antibodies produced similar measurements of C-bouton size on the control non-injured side (11.3 µm^3^ compared with the 11.7 µm^3^ observed on the control side on our BoNT/A experiments- unpublished observations) suggesting that contralateral C-boutons were not affected by the ipsilateral BoNT/A injections at this dosage. Extra control experiments performed later with a newer lot number of the VAChT antibody produced significantly less intense labelling in general making it invalid to apply the same threshold-based analysis with the same parameters to measure the C-boutons. Given that our AIS measurements are not affected by intensity but location of labelling and the same lot number of Ankyrin G was still available allowed us to confirm there were no effects of the BoNT/A injections on the AISs of contralateral motoneurones at this dose. This is consistent with findings that changes in excitability following BoNT/A are dependent on the degree of block^12^.

### Clinical implications

The possible functional effect of lengthening of the AIS following BoNT/A injection would also at first appear to have no functional consequence if the neuromuscular junction is blocked. It should be noted, however, that this block will not necessarily be complete or equally affect all synapses in the same motor unit. Furthermore, although full recovery can take 2–5 months^39^, there is evidence for partial recovery already at two weeks post injection along with sprouting of new nerve terminals, although these appear to be transitory^40^. Given that changes in AIS length are associated with changes in firing frequency^15^, we can hypothesise that an increase in firing frequency in response to the same inputs would increase the motor unit’s effectiveness in summating the twitches of muscle fibres from surviving or newly made functional connections.

Importantly, all the work to date on excitability changes following BoNT/A have focused on the initial time period (normally ~ 2 weeks) after injection when the block is maximal. Whether the central excitability changes along with the anatomical changes that we have observed would return to normal upon full restoration of functional synaptic activity will be important to determine. Whilst a full restoration of central excitability cannot be discounted, neither can it be assumed. Similar, but more pronounced excitability changes are observed following axotomy and while most are returned to the pre-axotomy values after reinnervation of the muscle, not all are, including lasting impairments in proprioceptive inputs to the motoneurones^19,41^. In addition, only the responses to single doses of BoNT/A have been investigated in the current study and so it will be important in the future to investigate the long-term consequence of repeated injections. This is highly relevant as BoNT/A is increasingly being recommended for the treatment of a number of clinical disorders in which increased neuronal excitability is involved including idiopathic over-active bladder^42^, hemifacial spasms, cervical dystonia, various dyskinesias and spasticity following traumatic brain injury (reviewed by^43^) and even for conditions such as migraine^44^ and chronic pain^45^. BoNT/A is also currently being used in younger patients, most notably in children with cerebral palsy^46^. It will therefore be crucial to determine if the changes we have observed (and the corresponding excitability changes) are irreversible.

## Methods

### Animals and experimental design

The experimental procedures were approved by the Danish Animal Experiments Inspectorate (2014-15-0201-00442 and 2015-15-0201-00689) and were in accordance with the EU Directive 2010/63/EU.

### Botulinum toxin experiments

In 7 adult male rats (Wistar, 430–480 g) under isoflurane anaesthesia and aseptic conditions, the retrograde tracer, Cholera toxin subunit B (CTB, Thermo Fisher Scientific), conjugated to Alexa Fluor 488, was injected bilaterally into gastrocnemius muscle (0.05% in 10 µl Phosphate Buffered Saline, PBS). On one side, however, the CTB was mixed with 4IU BoNT/A (Botox®, Allergan) before injection. The side injected with CTB only served as an internal control. In one half of the rats the BoNT/A was injected on the right side and on the left side in the remainder.

The specific dosage of BoNT/A was selected to achieve a significant block whilst reducing confounds related to higher doses. Dose-response curves from another study using injections of BoNT/A into the whole rat triceps surae from 3IU up to 18IU show a 70% reduction in muscle force with dosages of only 3IU^47^. This went up to 76% at 6IU but with little additional change between this and 12IU units. Increasing dosages from 6 to 12IU, however, did result in an increasing effect on the contralateral limb (which in the current experiment we wanted to use as a control). Most importantly, the dosage we selected has previously been shown to result in enhanced stretch reflexes involving gastrocnemius motoneurones in rats at two weeks post injection^9,10^.

For pain management buprenorphine (Temgesic 0.3 mg/ml, Indivior UK Ltd, 0.03 mg/kg) was administered subcutaneously at the start of the procedure along with antibiotics (trimethoprim and sulphadiazine; Tribrissen, MSD Animal Health, Holland, 24%, 0.25 ml single dose) and buprenorphine was then administered post operatively for 3 days (Buprenorphine 2care4, Salutas Pharma GmbH, dose 0.4 mg/kg mixed with Nutella^TM^ for oral intake). Two weeks after the injections the rats were transcardially perfused with 4% paraformaldehyde in phosphate buffer (pH 7.4).

### Measurements of CMAPs

In 3 separate rats (Wistar, male, 395–410 g) BoNT/A was injected bilaterally into the gastrocnemius muscle but only on one side was the BoNT/A mixed with the tracer. The maximal CMAP was obtained before and 4 days after injection on both sides (under isoflurane anaesthesia). To do this, the sciatic nerve was stimulated using bipolar stimulating electrodes, increasing the intensity until the maximal CMAP was measured. This was measured using custom made silver ball electrodes that could be placed on the surface of the skin directly over the muscle. The signals were amplified and filtered using custom-made amplifiers (University of Copenhagen) and sampled and analysed using a 1401 digital to analogue converter run by Signal software (both CED, UK). The maximum amplitude of the CMAP was measured peak to peak. In all rats a drop of at least 75% amplitude was observed on both sides confirming that the BoNT/A did not impair the functional ability of the BoNT/A.

### Immunohistochemistry

After perfusion, a laminectomy was performed and the lumbar enlargement of the spinal cord was removed. The lumbar cord was sectioned into 50 µm thick horizontal sections and the sections containing traced motoneurones were selected for immunohistochemistry. For AIS labelling an antibody against Ankyrin G (rabbit anti-AnkG, 1:500, H215, Santa Cruz Cat no: sc28561) was used and for C-bouton labelling an antibody against the vesicular acetylcholine transporter was used (goat anti-VAChT, 1:500, Millipore, lot no. 2778849, ABN100). Immunohistochemistry was performed on free-floating horizontal sections. The tissue was first incubated in a blocking solution (5% normal donkey serum in PBST (phosphate buffered saline with 0.3% triton X-100) for 4 hours followed by overnight incubation with the primary antibodies diluted in blocking solution. The tissue was then sequentially incubated in the dark for 2 hours for each secondary antibody - donkey anti-rabbit Alexa Fluor 568 (Thermo Fisher Scientific Cat no: A-11055, 1:1000) and donkey anti-goat DyLight 650 (Invitrogen, lot nr. SA2325947, 1:1000). Sections were then rinsed, mounted and cover-slipped with fluorescent mounting medium (Dako Denmark).

### Image analysis

Tissue sections were imaged in Z-stacks with a Zeiss LSM700 confocal microscope, using a 20×/0.8 Plan Apochromat Air objective. For the AIS, image analysis was performed using ZEN 2.3 Black Software, and the following measurements were made: length of the AIS, distal and proximal width of the AIS, distance from the base of the axon hillock to the proximal end of the AIS and the 2D soma size calculated as an ellipse using the maximal and minimal diameter. Data from cells with an area of less than 600 µm^2^ and with no C-boutons on the soma were excluded as they are presumed to be γ-motoneurones^48–52^. AISs originating from dendrites were also excluded.

C-bouton measurements were performed using a semi-automated script on Image J (NIH Software). All images were pre-processed using Background substraction and Gaussian blur (sigma = 1). Number and size (volume and surface area) of C-boutons was measured using the 3-D Object counter function. C-boutons were measured from cells which had somas entirely contained in the image and not overlapping with other cells. The number of boutons counted was then confirmed manually, excluding C-boutons that were not on the soma and correcting for adjoining boutons that were measured as one. Furthermore, the middle section of each cell was determined manually and the perimeter and the area of the soma in the respective Z-stack were measured using Freehand selection tool in Image J. C-boutons around the centre perimeter were then identified using the script described above and counted manually.

All measurements were performed blinded and from the original raw data Z-stack files. Images for illustration were prepared using Image J (NIH Software) and Adobe Illustrator. Brightness and contrast were uniformly adjusted for image presentation using Image J.

### Statistics

Statistical tests were performed using the GraphPad Prism software. Precise details of the individual statistical tests are provided in the results and figure legends. All tests were two-tailed. D’Agostino & Pearson omnibus normality tests were used to test for normality and parametric statistics or non-parametric tests were then applied appropriately. Statistical significance was accepted at the P ≤ 0.05 level. On all graphs, stars are used to indicate the following significant differences: *(P ≤ 0.05), **(P ≤ 0.01), ***(P ≤ 0.001, ****(P ≤ 0.0001). On the graphs, unless indicated, no significant difference was found. Initial analysis compared individual cells from the 2 groups. Further analysis compared the means between the two sides by animal to confirm if any differences were consistent across animals, although caution must be used with the interpretation of these particular statistics due to low statistical power.

## Data Availability

The datasets generated and analysed during the current study are available from the corresponding author upon reasonable request.
